# Production of Gypenoside XVII from Ginsenoside Rb1 by Enzymatic Transformation and Their Anti-Inflammatory Activity In Vitro and In Vivo

**DOI:** 10.3390/molecules28197001

**Published:** 2023-10-09

**Authors:** Kailu Zhou, Yangyang Zhang, Yikai Zhou, Minghao Xu, Shanshan Yu

**Affiliations:** Northeast Asia Research Institute of Traditional Chinese Medicine, Changchun University of Chinese Medicine, Changchun 130117, China; zhoukl@ccucm.edu.cn (K.Z.); zhangyy@ccucm.edu.cn (Y.Z.); zhouyk@ccucm.edu.cn (Y.Z.); xumh@ccucm.edu.cn (M.X.)

**Keywords:** *Gynostemma pentaphyllum* (Thunb.) Makino, biotransformation, gypenoside XVII, β-glucosidase, anti-inflammation

## Abstract

The enzymatic transformation of the sugar moiety of the gypenosides provides a new way to obtain more pharmacologically active components. A gene encoding a family 1 glycosyl hydrolase from *Bifidobacterium dentium* was cloned and expressed in *Escherichia coli*. The recombinant enzyme was purified, and its molecular weight was approximately 44 kDa. The recombinant BdbglB exhibited an optimal activity at 35 °C and pH 5.4. The purified recombinant enzyme, exhibiting β-glucosidase activity, was used to produce gypenoside XVII (Gyp XVII) via highly selective and efficient hydrolysis of the outer glucose moiety linked to the C-3 position in ginsenoside Rb1 (G-Rb1). Under the optimal reaction conditions for large scale production of gypenoside XVII, 40 g ginsenoside Rb1 was transformed by using 45 g crude enzyme at pH 5.4 and 35 °C for 10 h with a molar yield of 100%. Furthermore, the anti-inflammatory effects of the product gypenoside XVII and its conversion precursor ginsenoside Rb1 were evaluated by using lipopolysaccharide (LPS)-induced murine RAW 264.7 macrophages and the xylene-induced acute inflammation model of mouse ear edema, respectively. Gypenoside XVII showed improved anti-inflammatory activity, which significantly inhibited the generation of TNF-α and IL-6 more effectively than its precursor ginsenoside Rb1. In addition, the swelling inhibition rate of gypenoside XVII was 80.55%, while the rate of its precursor was 40.47%, the results also indicated that gypenoside XVII had better anti-inflammatory activity than ginsenoside Rb1. Hence, this enzymatic method would be useful in the large-scale production of gypenoside XVII, which may become a new potent anti-inflammatory candidate drug.

## 1. Introduction

*Gynostemma pentaphyllum* (Thunb.) Makino (GpM), also known as “Jiao-Gu-Lan”, has been used as a precious medicinal herb in China and its application has been documented as early as the Ming Dynasty [1]. *G. pentaphyllum* has been evidenced to possess diverse pharmacological effects, such as anti-tumor [2], anti-inflammation [3], anti-virus [4], anti-atherosclerosis [5], anti-oxidation [6], anti-hyperglycemia [7], and neuroprotective activities [8].

Gypenosides, as the major bioactive constituents of *G. pentaphyllum*, are compounds with dammarane triterpenoid structures [9]. It is worth noting that *G. pentaphyllum* is also called “south ginseng” or “cheaper ginseng” because gypenosides are structurally similar to ginseng saponins, also called ginsenosides. Noticeably, some ginsenosides, which are characteristic saponins for *Panax* species, are also isolated from *G. pentaphyllum*, such as ginsenoside Rb1, Rb3, Rd, Rg3, Rg5, (20S and R)-Rh1, F1, F2, Rc, and (20S and R)-Rg2. Until now, more than 300 gypenosides with diverse structures have been characterized [9]. The structural diversity is based on the variety of aglycone (sapogenin) and glycone (sugar chains). The basic aglycone is protopanaxadiol, which is of dammarane type triterpenoid skeleton. The dammarane-type scaffold is a tetracyclic triterpenoid, which is characterized by a β-methyl at C-8, β-H at C-13, α-methyl at C-14, and β side chain at C-17. More importantly, attention has been paid to the study of sugar chains in the structure of *G. pentaphyllum.* It has been demonstrated that structural differences such as linked positions, numbers, and types of sugar moieties in gypenosides contribute to their different pharmacological activities. In addition, gypenosides with fewer sugar moieties are proven to be more pharmaceutically active. For instance, Zheng et al. reported that gypenoside TN-1 deglycosylated from gypenoside XLVI by using naringinase showed significantly higher inhibitory effect on SMMC7721 and Bel7402 hepatoma cells than the glycosylated precursor [10]. Cui et al. also demonstrated that gypenoside LXXV, which was one of the deglycosylated shapes of Gyp XVII, significantly reduced cell viability and displayed an enhanced anti-cancer effect compared to Gyp XVII [11]. Hence, the structural transformation of the sugar moiety of the gypenosides provides a new way to obtain more pharmacologically active components.

Enzymatic transformation is the use of enzymes to modify the specific molecular structure of naturally active compounds to obtain efficient and new compounds with low toxicity. Compared to chemical transformation, enzymatic biotransformation has the following advantages: (1) High selectivity, including substrate selectivity, stereoselectivity, and regioselectivity. It can undergo steric modification, thereby avoiding the protection of specific functional groups of naturally active compounds, moreover, unprotection steps can reduce the complexity of the process. (2) High efficiency. Biocatalysts can efficiently catalyze specific reactions with high product yields. (3) Environmentally friendly. Biotransformation reactions are mostly carried out at room temperature and in neutral environments, thereby reducing product decomposition, isomerization, racemization, and rearrangement reactions. The biotransformation reaction produces less waste and reduces environmental pollution. In addition, biological transformations can also complete some reactions that are difficult to have undergo chemical transformation. Until now, enzymatic transformation of gypenosides has been attempted, especially for the deglycosylation reaction of sugar moieties in gypenosides. For instance, β-glucosidase from *Lactobacillus brevis* was used for the biotransformation of Gyp XVII to CK [12]. In our previous work, a thermophilic glycoside hydrolase from *Fervidobaterium pennivorans* DSM9078 was identified to transform gypenoside XLIX into gylongiposide I via highly selective and efficient hydrolysis of the glucose moiety linked to the C21 position in gypenoside XLIX [13]. However, the number of gypenoside-transforming enzymes is still limited. Additionally, the thermostability, transformation activity, and specificity of most enzymes still do not meet the industrial demands. It is therefore meaningful to explore novel gypenoside-transforming glycosidases with good thermostability, high catalytic efficiency, and specificity.

Inflammation is a non-specific heterogeneous response of organisms to various stimuli (such as infection, injury, allergies, etc.), typically manifested as symptoms of local tissue redness, swelling, heat pain, and dysfunction. During the inflammatory process, the body releases a series of pro-inflammatory cytokines through inflammatory cells (including macrophages, monocytes, and neutrophils), causing tissue pain and damage [14]. Pro-inflammatory cytokines include tumor necrosis factor-α (TNF-α), inducible nitric oxide synthase (iNOS), nitric oxide (NO), cyclooxygenase-2 (COX-2), interleukin-1β (IL-1β), interleukin-6 (IL-6), interleukin-12 (IL-12), and interferon-γ (IFN-γ) [15]. Excessive production of inflammatory mediators can lead to many diseases, such as arthritis, septic shock, and inflammatory bowel disease [16]. Gypenosides exhibit strong suppression on NO production and iNOS expression in LPS-induced RAW 264.7 macrophages without interfering cell viability [17,18]. In a dextran sulfate sodium (DSS)-induced acute colitis mouse model, the expression of pro-inflammatory cytokines (IL-6, IL-12, IFN-γ, and TNF-α) from cells treated with gypenosides is significantly decreased [15]. The decreased levels of inflammatory mediators (IL-6, IL-1β, COX-2, TNF-α, and NO) are also observed in LPS-stimulated RAW 264.7 macrophage cells treated with gypenosides purified from tetraploid *G. pentaphyllum* leaf extract [3]. The inhibition of NF-κB activation of gypenosides is also observed in the optic nerve and retinal cells. Gypenosides are also found to attenuate LPS-induced demyelination and retinal ganglion cell damage [19]. Additionally, gypenosides are capable of inhibiting IL-1β in human osteoarthritis chondrocytes, leading to the reduction in NO and prostaglandin E2 (PGE2) production [20]. There have also been studies on anti-inflammatory mechanisms of pure compounds from *G. pentaphyllum*. Gypenoside IX is found to downregulate the production of inflammatory mediators (TNF-α, iNOS, IL-6, IL-1β, and COX-2) in LPS-stimulated C6 cells [21]. Some other gypenosides are also reported to exhibit potent in vitro anti-inflammatory activities [22,23,24,25]. Due to the diverse structure of gypenosides, further research on their anti-inflammatory activity is still needed, and it is expected that more active compounds from *G. pentaphyllum* with anti-inflammatory activity will be discovered.

Gyp XVII is one of the major active gypenosides in *G. pentaphyllum*. It accounts for 4.04% of the total saponins in *G. pentaphyllum*. Increasing evidence shows that Gyp XVII displays various pharmacological effects such as cardiac protection [26], anti-depression [27], and neuroprotection [28]. However, the anti-inflammatory activity of Gyp XVII has not been reported yet. Gyp XVII is a dammarane-style structure of tetracyclic triterpenes, which has one glucose located at the C-3 position and two glucoses connected to the C-20 position of the aglycone. Based on the chemical structure similarity between Gyp XVII and G-Rb1, the purpose of this study is to establish an effectively enzymatic method to produce Gyp XVII from G-Rb1, so as to lay a foundation for the pharmacological activity, research, and application development.

In our research, the large-scale biotransformation of Gyp XVII from G-Rb1 by using an enzymatic method was studied. Moreover, the anti-inflammatory effects of Gyp XVII and its precursor G-Rb1 were investigated through LPS-induced RAW 264.7 macrophages and xylene-induced acute ear edema models in mice. This enzymatic method would be useful in the large-scale production of Gyp XVII, which may become a new potent anti-inflammatory candidate drug.

## 2. Results

### 2.1. Sequence Analysis of BdbglB from B. dentium

**Figure 1:**
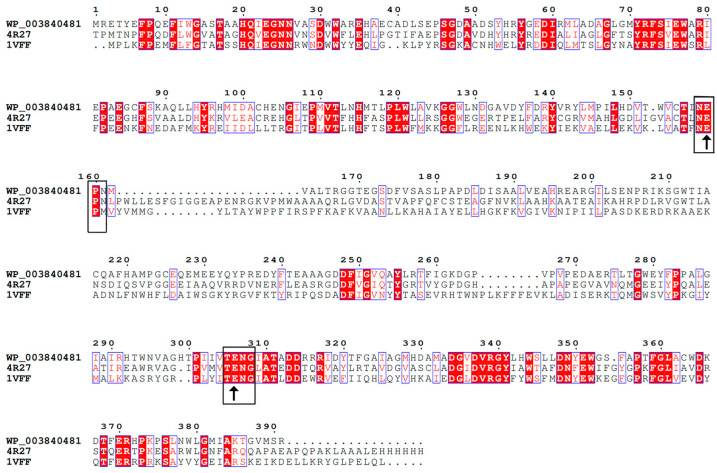
Multiple amino acid sequence alignment of BdbglB with characterized glycoside hydrolases from GH1. The accession numbers of the aligned sequences are for the following organisms: WP_003840481, BdbglB from *B. dentium*; 4R27, β-glycosidase from *Microbacterium* sp. Gsoil167; 1VFF, β-glycosidase from *Pyrococcus horikoshii*. The accession numbers were indicated to the left of the amino acid sequences. Identical residues are indicated by a red background. Symbols: ↑—amino acids forming a catalytic residue.

The family 1 glycosyl hydrolase gene BdbglB consisted of 1173 bp encoding 391 amino acids with a theoretical molecular mass of 43.89 kDa and a theoretical pI value of 4.78. The amino acid sequence of BdbglB (Genbank No. WP_003840481) exhibited the highest similarity with GH 1 proteins from *Bifidobacterium adolescentis* (92.3% identity, Genbank No. WP_195400497) and *Enterococcus durans* (91.8% identity, Genbank No. WP_090223359.1). These proteins have not yet been characterized. The alignment of BdbglB with the other two characterized glycoside hydrolases from GH1 indicated that these proteins shared some conserved peptide motifs, namely NEP (residues 158–160) and TENG (residues 305–308) (Figure 1). The Glu159 and Glu306 residues were typical catalytic residues of the GH1 enzymes, which confirmed that BdbglB belonged to the GH1 family. Moreover, the conserved domains of BglB and the glycosyl hydrolase superfamily 1 were retrieved in the protein sequence of BdbglB. Most of the β-glucosidases from the GH1 family contained the BglB domain which contained genes that encoding phospho-β-glucosidases.

### 2.2. Expression, Purification, and Characterization of BdbglB from B. dentium

**Figure 2:**
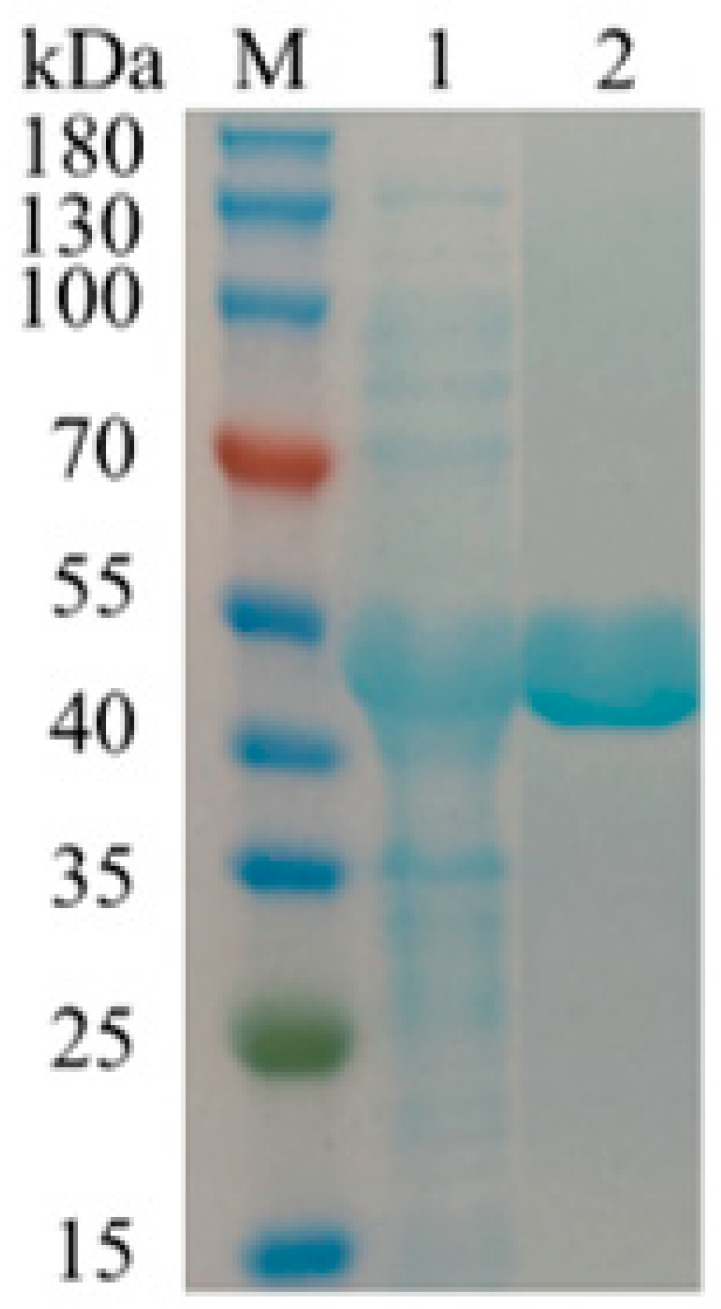
Purification of BdbglB. Lane M, molecular mass marker; Lane 1, crude enzyme solution before Ni–NTA affinity chromatography purification; Lane 2, pure enzyme solution BdbglB after Ni–NTA affinity chromatography purification.

The gene was cloned and expressed in *Escherichia coli* (*E. coli*) under the control of the IPTG-inducible promoter T7. After being induced under 16 °C for 12 h with 1 mM IPTG, the recombinant enzyme was solubly overexpressed in *E. coli* cells. The recombinant BdbglB was purified by His-trap affinity chromatography with a final purification of 1.1-fold and a specific activity of 99 U/mg for *p*-nitrophenyl β-d-glucopyranoside (*p*-NPG). The expressed enzyme was determined as a single band by SDS-PAGE, with a molecular mass of approximately 44 kDa (Figure 2), which was almost consistent with the molecular weight of 43.89 kDa calculated from 391 amino acids.

**Figure 3:**
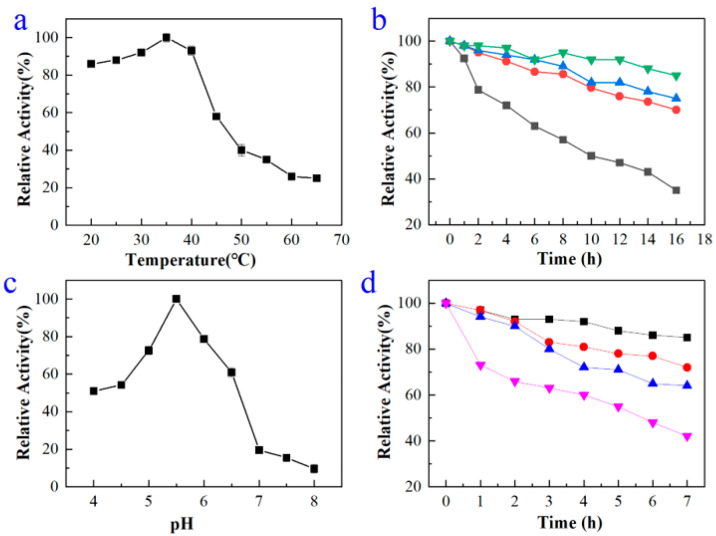
(**a**) Effect of temperature on enzyme activity. (**b**) Effect of temperature on enzyme stability. The activities were determined by assays with *p*-NPG as the substrate following incubation of the enzyme at 25 °C (▼), 35 °C (▲), 45 °C (●), and 55 °C (■) for the indicated times. (**c**) Effect of pH on enzyme activity. (**d**) Effect of pH on enzyme stability. The activities were determined by assays with *p*-NPG as the substrate following incubation of the enzyme at pH 4.4 (■), pH 5.4 (●), pH 6.4 (▲), and pH 7.4 (▼) for the indicated times.

The results of the optimal temperature and thermal stability of recombinant enzyme BdbglB were shown in Figure 3a,b, respectively. The optimal temperature for BdbglB to catalyze substrate *p*-NPG was 35 °C, which was a relatively moderate catalytic temperature. When the temperature was between 20 and 40 °C, the catalytic activity of BdbglB remained at a high level, both exceeding 80%. It can be seen that this enzyme belonged to mesophilase [29]. Moreover, the thermostability of BdbglB was studied by incubating the enzyme in 50 mM phosphate-citrate buffer (pH 5.4) for various lengths of time at 25, 35, 45, and 55 °C, and then the residual activities were measured on *p*-NPG. Activity of the enzyme obtained at 0 h was defined as the control (100% activity). After 10 h of incubation at 25, 35, and 45 °C, respectively, the enzyme exhibited above 80% relative activity, however, at 55 °C after 10 h, the residue activity dropped to about 50% relative activity.

The results of the optimal pH and pH stability of recombinant enzyme BdbglB were shown in Figure 3c,d, respectively. The optimal pH for BdbglB to catalyze substrate *p*-NPG was 5.4. The pH stability profiles were determined by incubating the enzyme in 50 mM phosphate-citrate buffer solutions (pH 4.4, pH 5.4, pH 6.4, and pH 7.4) at 35 °C for various lengths of time. Activity of the enzyme obtained at 0 h was defined as the control (100% activity). After being incubated for 2 h in pH 4.4, pH 5.4, and pH 6.4 buffer solutions, respectively, the enzyme exhibited about 90% relative activity. While, in pH 7.4 buffer solution after 2 h, the residue activity dropped to below 70% relative activity, indicating that BdbglB was more stable at acid pHs (Figure 3d).

Table 1 showed the effects of different metal ions and EDTA on the activity of the recombinant enzyme BdbglB. At the concentration of 5 mM, Na^+^ was able to slightly increase enzyme activity. In contrast, NH_4^+^_, K^+^, and Zn^2+^ were able to slightly inhibit enzyme activity. Moreover, Mn^2+^ significantly increased BdbglB activity, while Ca^2+^, Ba^2+^, Fe^3+^, and Co^2+^ significantly inhibited BdbglB activity. Except for NH_4^+^_ and Mn^2+^, the promoting or inhibitory effects of other metal ions on enzyme activity increased with the increase in ion concentration. Interestingly, NH_4^+^_ and Mn^2+^ exhibited opposite effects at low and high concentrations. Mn^2+^ exhibited significant promoting effects at low concentrations. However, as Mn^2+^ concentration increased to 10 mM, Mn^2+^ had a slight inhibitory effect on BdbglB activity. The chelating agent EDTA had almost no inhibitory activity, which indicated that BdbglB was not a metalloprotein.

The substrate specificity of BdbglB was investigated using substrates including *p*NPβGlu, *p*NPαGlu, *p*NPαAraf, *p*NPαArap, *p*NPβLac, *p*NPβGal, *p*NPβMan, *p*NPαMan, *p*NPβXyl, *p*NPβFuc, *p*NPβCel, and *p*NPαRha (Table 2). The purified enzyme BdbglB displayed the highest activity towards *p*NPβGlu. In addition, BdbglB was also active on *p*NPβGal, *p*NPβMan, and *p*NPβXyl, but undetectable activity on other substrates, indicating that it was an β-glucosidase.

### 2.3. Structural Analysis of the Biotransformed Product of G-Rb1

**Figure 4:**
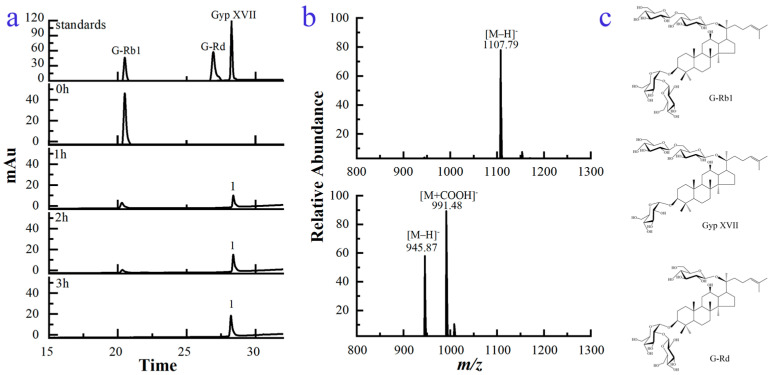
(**a**) HPLC analysis of G-Rb1 during the biotransformation process using the recombinant enzyme BdbglB. The standards were indicated on the peaks. Number was used to indicate the product peak. (**b**) MS spectra of G-Rb1 (**up**) and its transformed product (**down**). (**c**) Structural characteristics of G-Rb1, Gyp XVII, and G-Rd.

The biotransformation process of G-Rb1 by using the recombinant enzyme was studied via high performance liquid chromatography (HPLC). As indicated in Figure 4a, G-Rb1 had a retention time of 20.539 min. After transformation for 1 h, its peak area decreased dramatically, and one new peak 1 was observed at the retention time of 28.277 min. G-Rb1 was completely converted after 3 h. Additionally, no more transformed product was observed with the extension of reaction time.

As seen in Figure 4c, G-Rb1 possessed two glucoses linked on the C-20 position and two glucoses attached on the C-3 position of PPD-aglycone. The molecular weights of G-Rb1 and the transformed product were tentatively investigated by high performance liquid chromatography-tandem mass spectrometry (HPLC/MS) (Figure 4b). The molecular weight of G-Rb1 was calculated to be 1109 based on its [M−H]^−^ ion at *m*/*z* 1108, corresponding to a molecular formula of C_54_H_92_O_23_. Similarly, the molecular weight of the transformed product was determined to be 947 based on its [M−H]^−^ ion at *m*/*z* 946 and [M+HCOO]^−^ ion at *m*/*z* 991, corresponding to a molecular formula of C_48_H_82_O_18_. The mass difference between 1109 and 947 was 162, which corresponded to a glucose moiety. According to the structure of G-Rb1, the compound with a molecular weight difference of one glucoside could only be ginsenoside Rd (G-Rd) or Gyp XVII. G-Rd and Gyp XVII were isomers (Figure 4c). G-Rd had one glucose linked on the C-20 position and two glucoses attached on the C-3 position of PPD-aglycone. Gyp XVII possessed two glucoses linked on the C-20 position and one glucose attached on the C-3 position of PPD-aglycone. Moreover, based on the analysis results of HPLC (Figure 4a), the retention times of standard G-Rd and standard Gyp XVII were 26.936 min and 28.277 min, respectively. The retention time of the transformed product (peak 1) was consistent with that of standard Gyp XVII, so it was further identified as Gyp XVII. The structural difference between G-Rb1 and Gyp XVII lied only in the number of glucose residues at the C-3 position of the aglycone. Hence, Gyp XVII was generated by hydrolysis of one outer glucose residue at the C-3 position of G-Rb1. Since no other enzymes were introduced into the reaction system, we believe that the conversion of Gyp XVII from G-Rb1 was a glucoside hydrolysis reaction catalyzed by recombinant glycoside hydrolase BdbglB from *B. dentium*.

**Figure 5:**
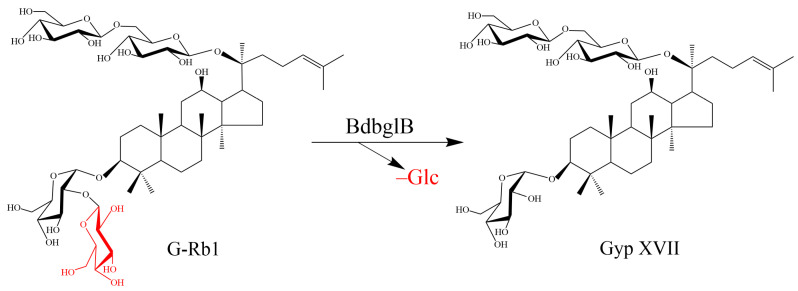
The biotransformation pathway of G-Rb1 by using the recombinant enzyme BdbglB.

The biotransformation pathway was illustrated in Figure 5, G-Rb1 was transformed into Gyp XVII by using the recombinant enzyme BdbglB via the hydrolysis of the outer glucose moiety at the C-3 position of the aglycone.

### 2.4. Large-Scale Production of Gyp XVII by Using the Recombinant Enzyme BdbglB

**Figure 6:**
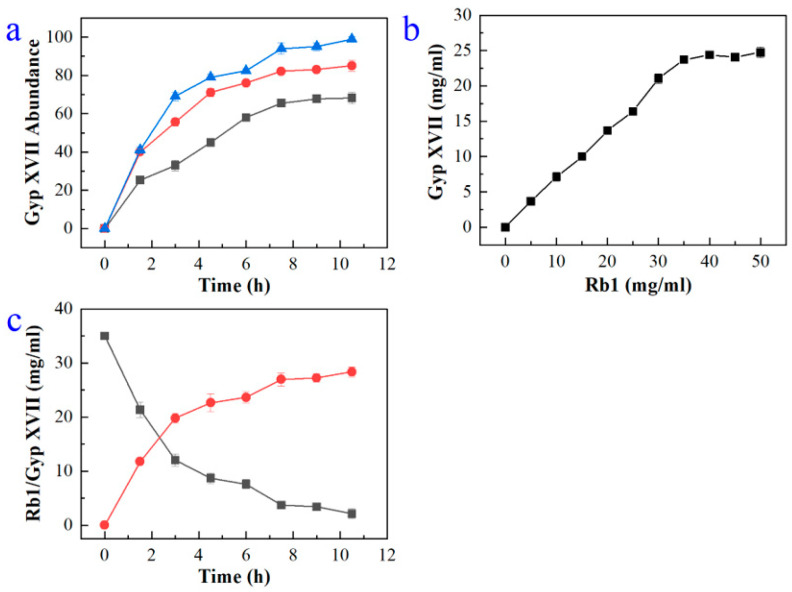
(**a**) Effect of the concentrations of crude enzyme including 25 mg/mL enzyme (■), 35 mg/mL enzyme (●), and 45 mg/mL enzyme (▲) on Gyp XVII production by using the recombinant enzyme BdbglB. (**b**) Effect of the concentrations of G-Rb1 on Gyp XVII by using the recombinant enzyme BdbglB. (**c**) Effect of the transformation time on Gyp XVII production (●) from G-Rb1 (■) by using the recombinant enzyme BdbglB.

In order to reduce production costs, the effect of crude enzyme concentration on Gyp XVII production was investigated with 25 mg/mL G-Rb1 as the substrate by varying the enzyme concentration from 25–45 mg/mL. Gyp XVII production increased with increasing the enzyme concentration. The conversion of G-Rb1 reached 100% using 45 mg/mL enzyme within 10 h (Figure 6a), indicating that the enzyme concentration was optimal at 45 mg/mL.

In consideration of decreasing the reactor volume, the effect of substrate concentration on Gyp XVII production was assessed by varying the concentration of G-Rb1 from 0 to 50 mg/mL and reacting with 45 mg/mL enzyme. The Gyp XVII production increased with increasing G-Rb1 concentration and reached a plateau above 40 mg/mL (Figure 6b). Thus, the optimal substrate concentration was determined to be 40 mg/mL.

In order to determine the most appropriate reaction time, the time course of the biotransformation of G-Rb1 to Gyp XVII was monitored via HPLC analysis. As seen in Figure 6c, the production of Gyp XVII reached its highest level after 10 h of biotransformation, and the Gyp XVII was transformed almost completely.

Hence, the scaled-up biotransformation was performed in a 5.0 L glass bottle (2.0 L working volume) under optimal conditions (shaking 200 rpm for 10 h at pH 5.4 and 35 °C). The crude recombinant enzyme (45 mg/mL) was reacted with an equal volume of G-Rb1 (40 mg/mL). Under the optimal reaction conditions, G-Rb1 was transformed to Gyp XVII with a molar yield of 100%.

### 2.5. Effects of Gyp XVII and Its Precursor G-Rb1 on LPS-Stimulated RAW 264.7 Cell

**Figure 7:**
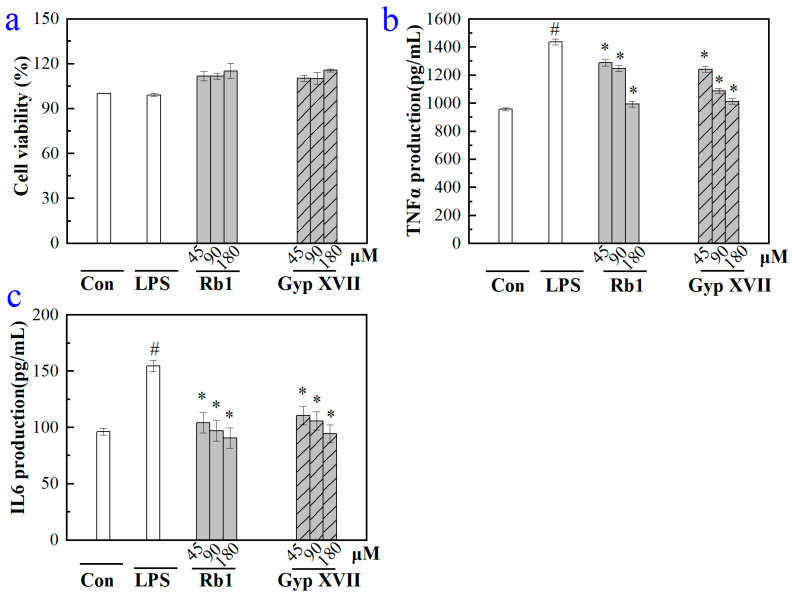
(**a**) Effects of G-Rb1 and Gyp XVII on cell viability of RAW 264.7 cells. RAW264.7 cells are incubated with G-Rb1 and Gyp XVII at indicated concentrations for 24 h. Cell viability was measured by CCK-8 assay. The data are means ± SD (*n* = 3). *p* > 0.05 vs. the blank control. (**b**,**c**) Effects of G-Rb1 and Gyp XVII on productions of pro-inflammatory cytokines TNF-α and IL-6 in LPS-stimulated RAW 264.7 cells, respectively. The data are means ± SD (*n* = 3). ^#^ *p* < 0.05 vs. the LPS-free control; * *p* < 0.05 vs. the LPS treated group.

Evaluation of the effects of Gyp XVII and its precursor G-Rb1 on RAW264.7 mouse macrophage cells viability was conducted using CCK-8 assay. As depicted in Figure 7a, following 24-h treatment, Gyp XVII and G-Rb1 at concentrations ranging from 45 to 180 μM had no effect on RAW264.7 cell viability. Hence, the concentration range of saponins can be used for further measurements.

Pro-inflammatory cytokines play a crucial role in the process of inflammation and related diseases. To investigate the anti-inflammatory activity at the cellular level, RAW 264.7 mouse macrophage cells stimulated by LPS (1 μg/mL) were used, and the content of pro-inflammatory cytokines TNF-α and IL-6 were determined. As noted in Figure 7b,c, the amount of TNF-α and IL-6 in the cell culture medium was markedly increased (1435 pg/mL and 155 pg/mL) in the model group subjected to 24 h LPS (1 μg/mL) treatment versus the control group that did not receive LPS stimulation. Compared with the model, treatment with G-Rb1 and Gyp XVII significantly inhibited LPS-induced TNF-α and IL-6 production in a dose-dependent manner, and the result indicated that G-Rb1 and Gyp XVII had great potential to improve the anti-inflammatory effects.

### 2.6. Effects of Gyp XVII and Its Precursor G-Rb1 on Mouse Ear Swelling Response

**Figure 8:**
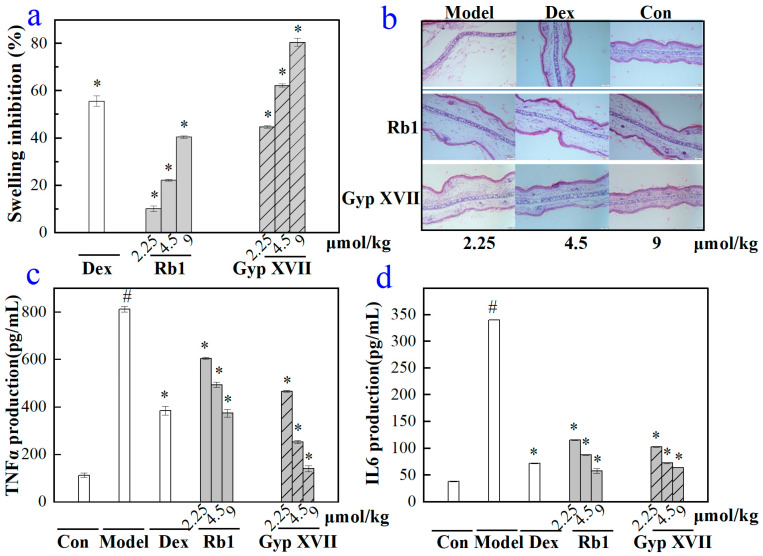
(**a**) Effects of Rb1 and Gyp XVII on the mouse ear swelling response. The bar chart represented ear swelling inhibition rate of the Dexamethasone group (1.5 mg/kg) and the saponins groups (2.25, 4.5, and 9 µmol/kg, respectively). * *p* < 0.001 indicated significant difference from vehicle control. (**b**) Histopathology analysis of the inhibitory effect of G-Rb1 and Gyp XVII on the xylene-induced inflammatory response in mouse ear swelling. Scale bars, 100 μm. Magnification, 200×. (**c**,**d**) Effects of G-Rb1 and Gyp XVII on the production of pro-inflammatory cytokines TNF-α and IL-6 in the xylene-induced ear swelling model. The data are means ± SD (*n* = 3). ^#^ *p* < 0.01 vs. the control group, indicated significant difference from dexamethasone-treated group. * *p* < 0.001 vs. the control group.

To evaluate the anti-inflammatory activity of G-Rb1 and GypXVII in vivo, a xylene-induced acute inflammation model of mouse ear edema was used. In the xylene-treated model group, the inflammation symptoms of swelling and redness in the right ear of mice were more obvious. As shown in Figure 8, mice pretreated with 2.25–9 μmol/kg G-Rb1, Gyp XVII, and the positive control drug dexamethasone showed significant reductions in ear swelling. Moreover, we also observed differences in the thickness of the perforated ear tissue structure (Figure 8b). In the groups treated with G-Rb1 and Gyp XVII, the thickness of the mouse ear tissue decreased in a dependent manner. Under the treatment of 9 μmol/kg of G-Rb1 and Gyp XVII, the mouse ear tissue basically returned to its normal structure.

In addition, we also validated the effects of G-Rb1 and Gyp XVII on the production of pro-inflammatory cytokines TNF-α and IL-6 in the xylene stimulated mouse ear swelling response (Figure 8c,d). In the xylene-induced model group, the concentration of pro-inflammatory cytokines TNF-α and IL-6 significantly increased to 812 pg/mL and 340 pg/mL, respectively. Compared with the model group, G-Rb1 and Gyp XVII could significantly inhibit the production of TNF-α and IL-6 stimulated by xylene, with a dose-dependent inhibitory effect. At the same time, we found that at high concentrations of 9 umol/kg, the inhibitory rate of Gyp XVII on mouse ear swelling was as high as 80.55%, while the inhibitory rate of G-Rb1 on mouse ear swelling was only 40.47% (Figure 8a). Hence, Gyp XVII had a more significant anti-inflammatory effect than G-Rb1.

## 3. Discussion

In recent years, enzymatic transformation has gradually become the main method to produce the rare active compounds in Chinese herbs on a large scale, owing to its strong specificity, mild conditions, and fewer byproducts. The main process of enzymatic transformation is through the hydrolysis of saponin glycosyl groups using glycosidases. Under the catalysis of glycoside hydrolases, a series of structural changes can occur and are mainly caused by the step-by-step desugar process, and the generated conversion products can have better bioavailability or stronger biological activity than the original saponins.

Gyp XVII, the main active compound in *G. pentaphyllum*, has various pharmacological effects including cardioprotection [3], neuroprotection [28], and anti-atherosclerosis [5]. However, due to the difficulty in obtaining a large amount of Gyp XVII, in-depth theoretical research and application development are still limited. Theoretically, Gyp XVII can be produced from G-Rb1 transformation as they have a similar nucleus structure, except for a difference in the number of glycosyl side chains. Therefore, the biotransformation of Gyp XVII has been attempted through enzymatic conversion. For example, Cui et al. found that the β-glucosidase from *Actinosynnema mirum* could achieve the conversion of G-Rb1 to Gyp XVII and Gyp LXXV [30]. Wang et al. identified a β-glucosidase from *Sphingomonas sp.* 2F2 which converts G-Rb1 into Gyp XVII to F2 [31]. In Table 3, the enzymatic transformations of Gyp XVII were summarized. Prominent issues were found such as poor selectivity, byproduct generation [30,31,32,33,34], and lack of quantitative research [14,35,36,37,38] in the glycosidases currently available for the production of Gyp XVII, thereby limiting its industrial production and application. Hence, searching for new enzymes for Gyp XVII production has become a current research hotspot.

*Bifidobacterium* is an important human intestinal probiotic, which can hydrolyze and metabolize oligosaccharides and produce small molecules beneficial for the human body, such as lactate, acetate, short-chain fatty acids, and propionate [39]. β-Glucosidase, the key enzyme required for the metabolism and homeostasis of *Bifidobacterium*, hydrolyzes oligosaccharides to produce glucose, which can be further used as an energy source for organisms [40]. Therefore, *Bifidobacterium* is a rich source of glycoside hydrolases. Two β-glucosidases were successfully purified from *Bifidobacterium breve* 203 [41]. Due to high purity, high yield, repeatability, large-scale production, and safety, recombinant proteins have broad application prospects in fields such as biomedicine, agriculture, and industry. Certain β-glucosidases from *Bifidobacterium*, such as *Bifidobacterium longum* subsp. *Infantis* ATCC15697 [42], *Bifidobacterium adolescentis* ATCC15703 [43], *Bifidobacterium breve* 203, and *Bifidobacterium pseudocatenulatum* IPLA 36007 [44] can be expressed in *Escherichia coli* as soluble proteins. Previously reported glycosidases derived from *Bifidobacterium* mostly belong to the glycoside hydrolase family 3 [42,43,45]. In this study, a new GH1 glycosidase gene from *Bifidobacterium denium* was discovered and expressed solubly in *Escherichia coli*, thereby further enriching the glycosidase system derived from *Bifidobacterium* and expanding the application of this enzyme in the biotransformation of saponin.

β-Glucosidases belong to acidic proteins, and the optimal pH is generally within the acidic range, with little change, concentrated between 3.4 and 5.4. The optimal pH for BdbglB to catalyze substrate *p*-NPG was 5.4. In this study, when the pH value was greater than 6.4, the enzyme activity significantly decreased. This might be because the enzyme played a catalytic role on the premise that the catalytic group needed to be maintained in the correct ionized state, and in catalytic reactions, catalytic glutamate needed to act as an acid. A higher pH value might make ion protonation unstable and hinder catalytic reactions, leading to a decrease in enzyme activity. This was also why most enzymes were more sensitive to pH.

The recombinant enzyme BdbglB could specifically hydrolyze outer glucose on the C-3 position of G-Rb1 to generate Gyp XVII. BdbglB had better substrate specificity than some enzymes that had also been reported to convert G-Rb1 to Gyp XVII. Poor substrate specificity of enzymes leads to the production of byproducts during the conversion process. For example, β-glucosidases from *Actinosynnema mirum, Sphingomonas* sp. 2F2 [31], *Terrabacter ginsenosidimutans* sp. Nov [32], and *Penicillium decumbens* [33] could convert Rb1 to Gyp XVII, however, the substrate selectivity during the production process was not specific, resulting in the production of other products such as a small amount of Gyp LXXV, F2, or CK. In addition, some enzymes had substrate selectivity similar to BdbglB, which could specifically convert G-Rb1 to Gyp XVII, such as β-glucosidases from *Sphingopyxis alaskensis* [14], *Fervidobaterium pennivorans* DSM9078 [35], and *Bifidobacterium adolescentis* ATCC15703 [36]. However, there was a lack of quantitative conversion data in the research results of those enzymes. Hence, our study utilized the advantage of recombinant enzyme BdbglB specific hydrolysis of outer glucose at the C-3 position in G-Rb1 to efficiently produce Gyp XVII, and conducted quantitative and scaled-up preparation research on it, thereby filling the current limitations of Gyp XVII industrial production.

## 4. Materials and Methods

### 4.1. Materials

*Escherichia coli* strains were incubated at 37 °C in Luria–Bertani (LB) medium (10 g/L tryptone, 5 g/L yeast extract, and 10 g/L NaCl) with 50 mg/L kanamycin. Authentic G-Rb1 and Gyp XVII were purchased from Shanghai Yuanye Biological Technology Co. Ltd. (Shanghai, China). The *p*-nitrophenyl β-d-glucopyranoside (*p*NPβGlu), *p*-nitrophenyl α-d-glucopyranoside (*p*NPαGlu), *p*NP-α-l-arabinofuranoside (*p*NPαAraf), *p*NP-α-l-arabinopyranoside (*p*NPαArap), *p*-nitrophenyl β-d-lactose (*p*NPβLac), *p*-nitrophenyl β-d-galactose (*p*NPβGal), *p*-nitrophenyl β-d-mannose (*p*NPβMan), *p*-nitrophenyl α-l-mannose (*p*NPαMan), *p*-nitrophenyl β-d-xylose (*p*NPβXyl), *p*-nitrophenyl β-d-fucose (*p*NPβFuc), *p*-nitrophenyl β-d-cellobioside (*p*NPβCel), *p*NP-α-l-rhamnopyranoside (*p*NPαRha), and carboxymethyl cellulose (CMC) were purchased from Sigma-Aldrich (St. Louis, MO, USA). Acetonitrile, methanol, and formic acid of HPLC grade were obtained from Fisher Scientific (Waltham, MA, USA).

The RAW264.7 cell line was purchased from the Type Culture Collection of the Chinese Academy of Sciences (Shanghai, China). Dulbecco’s modified Eagle’s medium (DMEM) was obtained from Gibco (Carlsbad, CA, USA). Lipopolysaccharides (LPS) were purchased from Sigma (St. Louis, MO, USA). A Cell Counting Kit-8 (CCK-8) and enzyme-linked immunosorbent assay (ELISA) kit were obtained from Bestbio (Shanghai, China) and R&D (Minneapolis, MN, USA).

### 4.2. Sequence Analysis of BdbglB from B. dentium

Physicochemical properties of BdbglB, including amino acid quantity (aa), theoretical isoelectric point (PI), and relative molecular weight (MW) were analyzed on the ExPasy server (http://web.expasy.org/compute_pi/) (accessed on 30 June 2022) [46]. Homologs of BdbglB (Genbank No. WP_003840481) were searched with the BLASTp program on NCBI (https://blast.ncbi.nlm.nih.gov/Blast.cgi accessed on 30 June 2022). The glycoside hydrolases in GH1 family were chosen at the CAZy web (http://www.cazy.org/ accessed on 30 June 2022), and the sequence information of those proteins were collected by means of the CAZy web page links. Multiple alignments of BdbglB and the other two glycosidases from the GH1 family were performed using the ClustalX program (version 2.0) [47]. The Batch CD-search on the NCBI website was used to analyze the conserved domains of BdbglB, and the MEME program (version 5.1.0) was used to identify the conserved Motif.

### 4.3. Expression and Purification of BdbglB from B. dentium

The recombinant strains were collected by centrifugation at 6000× *g* for 20 min and resuspended in a lysis buffer (50 mM Tris-HCl, pH 7.1). The cells were then sonicated and centrifuged at 14,000× *g* for 30 min at 4 °C to remove the debris. The supernatants containing the target proteins were loaded onto a Ni-NTA affinity chromatography column (GE Healthcare) and purified using a 20–100 mM imidazole gradient. The purified enzyme was dialyzed against 50 mM phosphate-citrate buffer (pH 5.4) and concentrated to 1.5 mg/mL. The protein homogeneity was confirmed by 8% sodium dodecyl sulfate-polyacrylamide gel electrophoresis (SDS-PAGE).

### 4.4. Characterization of BdbglB from B. dentium

The *p*NP-β-D-glucopyranoside (*p*NPG) was used to test the β-glucosidase activity of BdgblB. Hydrolysis of *p*NPG was measured at 35 °C in 50 mM phosphate–citrate buffer (pH 5.4). The activity was determined by measuring the increase in absorbance at 405 nm due to the release of *p*NP. One unit (IU) of activity was defined as the amount of enzyme liberating 1 μmol of *p*-nitrophenol per min.

Using pNPG as the substrate, the effects of temperature and pH on enzyme activity were investigated by assaying the β-glucosidase activity according to the method described previously. The pH optima of BdbglB were tested over the pH range of 4.0–8.0 in 50 mM phosphate-citrate buffer at 35 °C. The temperature optima of BdbglB were measured between 20–65 °C (5 °C intervals) at the optimum pH.

Thermal stability of BdgblB was studied by incubating about 1.5 mg/mL purified enzyme solutions at 25 °C, 35 °C, 45 °C, and 55 °C for various lengths of time in 50 mM phosphate-citrate buffer (pH 5.4). The residual activity on *p*NPG was determined at 35 °C in 50 mM phosphate-citrate buffer (pH 5.4). Activity of the enzyme obtained at 0 h was defined as the control (100% activity).

The pH stability of BdgblB was tested by incubating about 1.5 mg/mL of purified enzyme solutions in pH 4.4, pH 5.4, pH 6.4, and pH 7.4 buffer solutions for various lengths of time at 35 °C. The residual activity on *p*NPG was determined at 35 °C in 50 mM phosphate-citrate buffer (pH 5.4). Activity of the enzyme obtained at 0 h was defined as the control (100% activity).

The effects of metal ions (Ba^2+^, Co^2+^, Mn^2+^, Ni^2+^, Mg^2+^, Na^+^, K^+^, Ca^2+^, Zn^2+^, Fe^2+^, and Hg^2+^) and EDTA on enzyme activity were investigated. The enzyme activity with no addition for the control was set as 100%. After incubation the enzyme with various metal ions and EDTA (5 mM and 10 mM) at room temperature for 30 min, the residual activity was measured according to the method described previously using *p*NPG as the substrate at pH 5.4 and 35 °C.

The substrate specificity of BdbglB was investigated by using the following substrates: *p*NPβGlu, *p*NPαGlu, *p*NPαAraf, *p*NPαArap, *p*NPβLac, *p*NPβGal, *p*NPβMan, *p*NPαMan, *p*NPβXyl, *p*NPβFuc, *p*NPβCel, and *p*NPαRha. For pNP glycoside substrates, 1 mL of reaction system includes *p*NP glycoside substrate with a final concentration of 5 mmol/L, buffer solution with a concentration of 50 mmol/L (pH 5.4), and 10 μg pure enzyme. The enzyme activities were measured according to the method described previously. One unit (IU) of activity was defined as the amount of enzyme liberating 1 μmol of p-nitrophenol per min.

### 4.5. Structural Analysis of the Biotransformed Product of G-Rb1

#### 4.5.1. HPLC Analysis

Chromatographic separation was performed using an UltiMate 3000 HPLC system (Thermo Fisher Scientific, Waltham, MA, USA) coupled with a Syncronis C18 chromatographic column (5 cm × 3.0 mm, 2.7 μm, Supelco, Bellefonte, PA, USA). The column oven temperature was maintained at 35 °C. Water and acetonitrile were used as the mobile phases A and B, respectively. The gradient elution was programmed as follows, with a flow rate of 0.5 mL/min: 0–10 min, 15–19% (B); 10–13 min, 19–25% (B); 13–18 min, 25–28% (B); 18–22 min, 28–30% (B); 22–25 min, 30–35% (B); and 25–30 min, 35–60% (B). The injection volume was set at 10 µL.

#### 4.5.2. HPLC-MS Analysis

Chromatographic separation was performed on the TSQ ENDURA triple quadrupole-liquid chromatography mass spectrometer (Thermo Fisher Scientific, Waltham, MA, USA) coupled with a Syncronis C18 chromatographic column (5 cm × 3.0 mm, 2.7 μm, Supelco, USA). The column oven temperature was maintained at 30 °C, and the mobile phases A and B were water with 0.1% formic acid aqueous solution and acetonitrile solution, respectively. The gradient elution program was determined as follows: 0–10 min, 15–19% (B); 10–13 min, 19–25% (B); 13–18 min, 25–28% (B); 18–22 min, 28–30% (B); 22–25 min, 30–35% (B); and 25–30 min, 35–60% (B). The injection volume was set at 5 µL with a flow rate of 0.3 mL/min. The scan range for MS acquisition was from 150–2000 *m*/*z* in negative ionization mode. Sheath gas flow was 35 L/min, auxiliary gas flow was 5 L/min, and capillary temperature was 350 °C.

### 4.6. Large-Scale Production of Gyp XVII from G-Rb1 by BdbglB from B. dentium

In order to optimize the enzyme and substrate concentrations, crude enzyme concentrations from 25 to 45 mg/mL (at 40 mg/mL G-Rb1) and substrate concentrations from 0 to 50 mg/mL (at 45 mg/mL enzyme) were evaluated. The time course reactions of Gyp XVII production were performed at 35 °C in 50 mM citrate/phosphate buffer (pH 5.4) containing 40 mg/mL G-Rb1, and 45 mg/mL crude enzyme. For all biotransformation reactions, samples were collected at regular intervals and HPLC was used to monitor the transformation process.

### 4.7. Assessment of Anti-Inflammatory Activity In Vivo and In Vitro

#### 4.7.1. Cell Culture and Treatment

RAW264.7 mouse macrophage cells were cultured in DMEM medium supplemented with 10% FBS, glutamine, and antibiotics at 37 °C under 5% CO_2_. Cells at 80–90% confluency were collected through centrifugation at 120× *g* at 4 °C for 10 min. The cell concentration was adjusted to 2 × 10^6^ cells/mL, and the cell viability was consistently greater than 90%. A 50-μL cell suspension was seeded into a tissue culture grade 96-well plate (4 × 10^5^ cells/well) and incubated for 2 h at 37 °C and 5% CO_2_ for cell attachment. Then, cells were stimulated by using 1 μg/mL of LPS with or without the presence of Gyp XVII and G-Rb1, and tested at the final volume of 100 μL/well. Cells were further incubated at 37 °C and 5% CO_2_ for 24 h for use.

#### 4.7.2. Cell Viability

Cell viability was determined using CCK-8 assay. RAW264.7 mouse macrophage cells were inoculated in a 96-well plate culture for 2 h, then divided into different groups: (1) equal volume culture medium as the control group. (2) Equal volume LPS (1 μg/mL) treatment for 24 h as the model group. (3) Equal volume Gyp XVII and G-Rb1 with different concentrations (45, 90 and 180 µM) treatment for 24 h as the drug group. There were three replicates per group. After treatment, the cells were mixed with CCK-8 (10 μL) and incubated in the dark for another 4 h. The absorbance at 450 nm was recorded using a PR 4100 microplate reader (Bio-Rad Inc., Hercules, CA, USA).

#### 4.7.3. Analysis of Cytokines Using ELISA Assay

TNF-α and IL-6 concentrations in the cell culture supernatant or the serum were detected using an ELISA kit according to the manufacturer’s instruction, and the results were expressed in pg/mL of protein. All of the analyses were performed in triplicate.

#### 4.7.4. Xylene-Induced Ear Swelling and Histological Analysis

All experiments involving the use of animals in this study were approved by the College of Basic Medical Sciences, Jilin University and performed in accordance with the animal welfare guidelines listed in the Guidelines for the Care and Use of Laboratory Animals. Fifty mice were divided into five groups (A–E) randomly, there was a vehicle control group (A) and positive controls (E), which were administered the same volume of physiological saline or dexamethasone (15 mg/kg), respectively. Groups B-D were administered Gyp XVII and G-Rb1 through tail vein injections at one of three different doses (2.25 µmol/kg, 4.5 µmol/kg, or 9 µmol/kg) for six days. In addition to the blank control group, the remaining mice in each group received a 20-μL smear of xylene on both the anterior and posterior surfaces of the right ear lobe at one hour after the sixth day of administration. Two hours later, blood was extracted from the eyeballs of mice and the mice were euthanized. The serum was collected by centrifugation at 3000 rpm for 10 min. The 9-mm sections were obtained from both ears with a cork borer, and the ear tissue was subsequently weighed. The degree of ear swelling was calculated according to the following formula: ear swelling inhibition rate (%) = (A − B)/A × 100, where A and B denoted ear swelling of the negative group and ear swelling of the drug groups, respectively. For histological analysis, ear biopsy samples were fixed with 4% paraformaldehyde, embedded in paraffin, and sectioned at a thickness of 9 mm. Sliced sections were stained with hematoxylin and eosin (H&E).

### 4.8. Statistical Analysis

Statistical analyses were performed with SPSS 13.0 software (SPSS Inc., Chicago, IL, USA). The data were analyzed by one-way ANOVA followed by Student’s two-tailed *t*-tests for comparison between two groups, and the data were presented as the mean ± SD. *p* < 0.05 indicated a statistically significant difference.

## 5. Conclusions

In the current study, a novel β-glucosidase was successfully cloned and expressed in *Escherichia coli*. The large-scale production of Gyp XVII from G-Rb1 was achieved by using the recombinant enzyme BdbglB with a molar yield of 100%. Moreover, Gyp XVII and G-Rb1 were proven to be novel, potent anti-inflammatory candidates. In the future, the ideal transformation properties of the recombinant β-glucosidase will make it a promising tool to produce more valuable deglycosylated saponins.

## Figures and Tables

**Table 1:** Effects of metal ions and EDTA on the enzyme activity.

Additives	Relative Activity (%)	
Control	100	100
Metal ions	5 mM	10 mM
NH_4_Cl	89.18	108.63
NaCl	109	119
BaCl_2_	68.28	33.91
KCl	90.34	93.83
MnCl_2_	135.66	93.81
CaCl_2_	63	28
ZnCl_2_	93.17	43.28
CoCl_2_	80.42	50.11
FeCl_3_	51.92	23.66
Inhibitors	5 mM	10 mM
EDTA	97.74	95.82

**Table 2:** Enzyme specificity for the enzyme BdbglB on various substrates.

Substrate	Relative Activity (%)
*p*NPβGlu	100 ± 0.87 ^a^
*p*NPαGlu	ND ^b^
*p*NPαAraf	ND
*p*NPαArap	ND
*p*NPβLac	ND
*p*NPβGal	7.5 ± 0.57
*p*NPαMan	ND
*p*NPβMan	6.4 ± 0.12
*p*NPβXyl	5.9 ± 0.85
*p*NPβFuc	ND
*p*NPβCel	ND
*p*NPαRha	ND

^a^ The activity against *p*NPβGlu was assumed to be 100% and corresponded to a specific activity of 99 U/mg. ^b^ Not detected, specific activity is not detected by the analytical methods used in this study.

**Table 3:** Summary of Enzymatic Biotransformation of Gyp XVII.

Organism	Reaction Conditions	GH Family	Substrate	Product	Reaction Time	Yield	Reference
*Sphingopyxis alaskensis*	pH 5.5, 50 °C	—	G-Rb1	Gyp XVII	7 h	87.5%	[14]
*Actinosynnema mirum*	pH 7.0, 37 °C	GH3	G-Rb1	Gyp XVII, Gyp LXXV	18 h	—	[30]
*Sphingomonas* sp. 2F2	pH 5.0, 37 °C	GH1	G-Rb1	Gyp XVII, F2	92 h	—	[31]
*Terrabacter ginsenosidimutans* sp. *nov.*	pH 7.0, 37 °C	GH3	G-Rb1	Gyp XVII, Gyp LXXV	24 h	—	[32]
*Penicillium decumbens*	pH 4.0, 60 °C	—	G-Rb1	Gyp XVII, F2, CK	13 h	—	[33]
*Arachidicoccus ginsenosidimutans* sp. *nov*.	pH 7.5, 50 °C	GH1	G-Rb1	Gyp XVII, F2, CK	4 h	—	[34]
*Fervidobaterium pennivorans* DSM9078	pH 5.5, 90 °C	GH5	G-Rb1	Gyp XVII	1 h	—	[35]
*Bifidobacterium adolescentis* ATCC15703	pH 7.0, 30–50 °C	GH1	G-Rb1	Gyp XVII	2 h	72.16%	[36]
*Leuconostoc mesenteroides* DC102	pH 6.0–8.0, 30 °C	—	G-Rb1	Gyp XVII	24 h	—	[37]
*Aspergillus oryzae*	pH 4.5, 50 °C	—	G-Rb1	Gyp XVII	1 h	88.75%	[38]

## Data Availability

Data are contained within the article.

## Sample Availability

Samples of the compounds gypenoside XVII and ginsenoside Rb1 are available from the authors.
